# Shaping with water: linking moisture perception to development in plant roots

**DOI:** 10.1186/s12915-026-02503-z

**Published:** 2026-01-17

**Authors:** William P. Dwyer, Héctor H. Torres-Martínez, José R. Dinneny

**Affiliations:** 1 Department of Biology, Stanford University, Stanford, CA 94305 USA; 2 Howard Hughes Medical Institute, Stanford, CA 94305 USA

**Keywords:** Water, Moisture, Roots, Hydropatterning, Hydrotropism, Xerobranching

## Abstract

Water is the most limiting resource for plant growth and development. Heterogeneity in the environmental distribution of water requires plants to direct root growth toward water and to avoid investing resources in areas that lack water. Roots use hydrosignaling pathways—hydrotropism, hydropatterning, and xerobranching—to sense and respond to water availability. While molecular mechanisms of water perception remain unclear, recent studies suggest that organ-level processes using proxies like ethylene help detect spatial water patterns. This review summarizes advances in hydrosignaling and identifies key knowledge gaps to address how plants sense water. Understanding these processes will guide strategies to improve root water capture for sustainable agriculture.

## Root responses to moisture

Since plants first evolved to live on land, navigating an environment with heterogeneous water availability has been a central feature of their environmental response repertoire. In soil, water is frequently non-uniformly distributed as a consequence of precipitation, gravity, and soil structure [1, 2]. Along the depth of a soil column, water typically flows down due to gravity but adheres to soil particles through matric effects. Soil texture and composition determine these matric effects as well as the size of pore spaces, which allow air to enter the rhizosphere and supply oxygen to respiring roots.

**Fig. 1:**
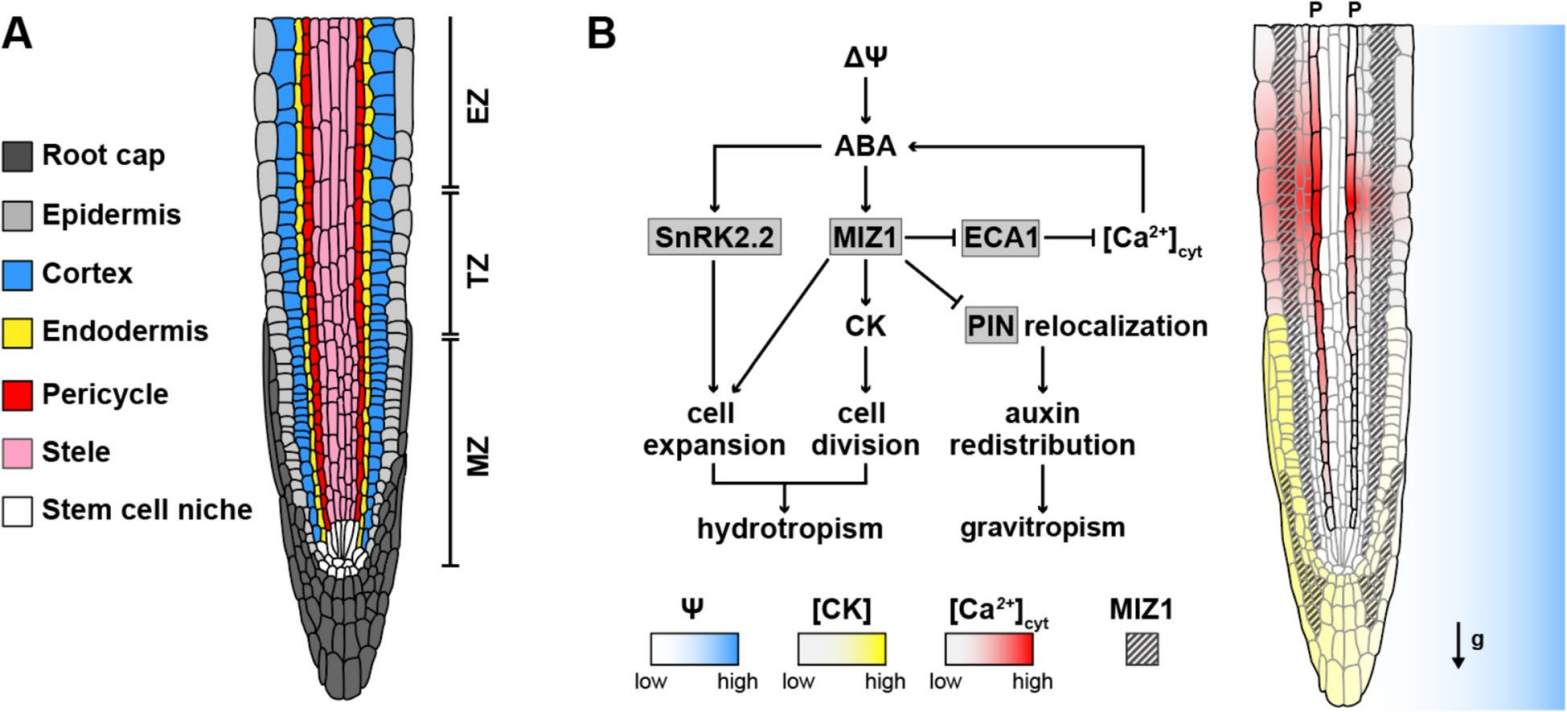
Molecular pathways for hydrotropism in the Arabidopsis root tip.** A** Root anatomy and developmental zones. Cell types are color coded. Meristematic (MZ), transition (TZ), and elongation (EZ) zones are indicated. **B** Unequal water potential (ψ) triggers ABA signaling and enhances cell elongation through MIZ1 activity in cortical tissue on the dry side [10]. ABA-dependent MIZ1 regulates asymmetric cytokinin (CK) distribution in the distal root tip, resulting in higher cell proliferation on the dry side [29]. MIZ1 antagonizes the gravitropic response through the inhibition of PIN-mediated auxin redistribution, in order to strengthen the hydrotropic response [30]. MIZ1 directly inhibits the endoplasmic reticulum Ca^2+^ ATPase (ECA1) to release Ca^2+^ into the cytosol, with a higher distribution on the dry side [21]. Cytosolic Ca.^2+^ travels through the phloem (indicated as P on top of the root) and into adjacent tissue layers. Root cross section adapted from De Smet et al. [35]

**Fig. 2:**
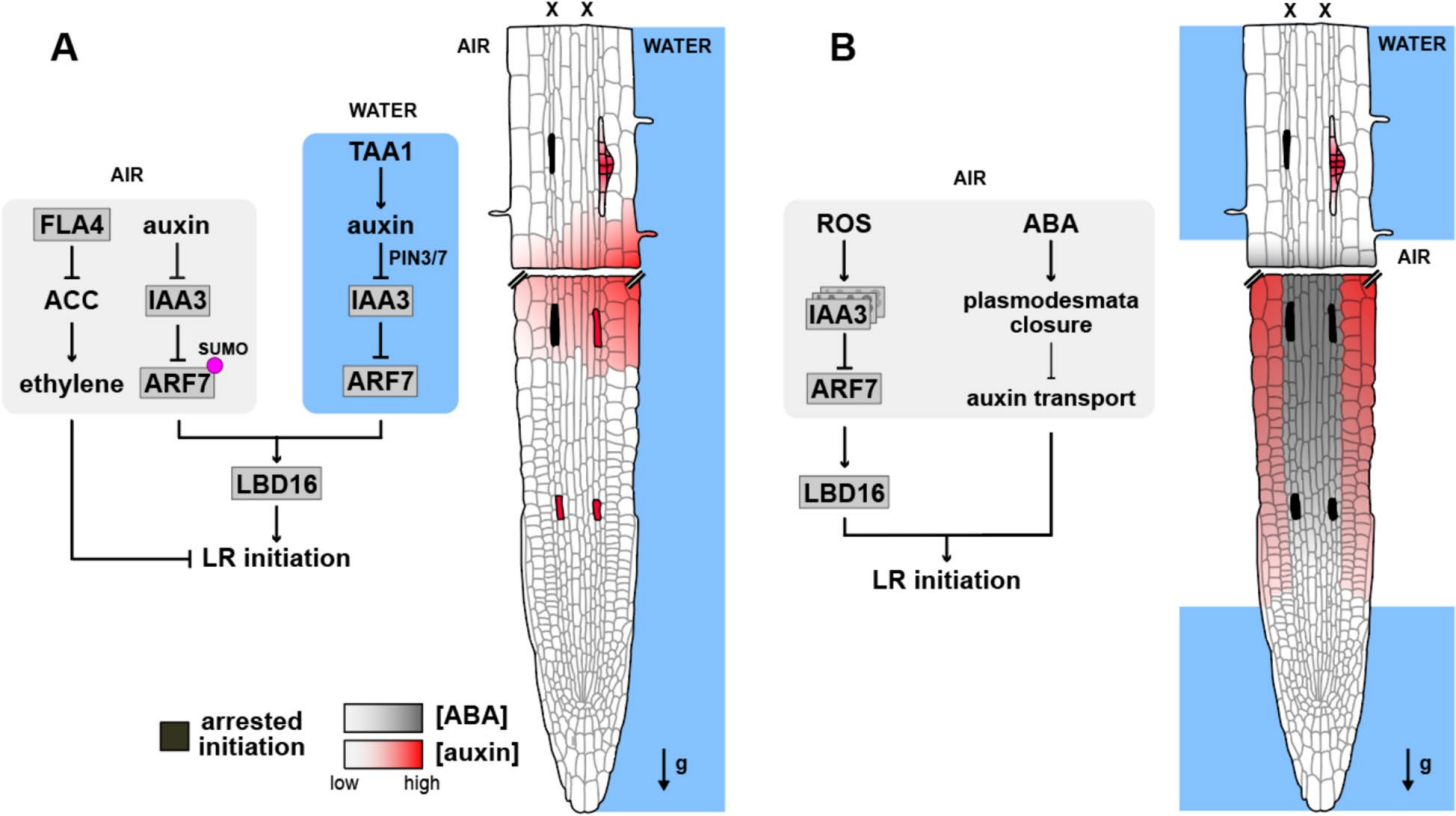
Molecular pathways for hydropatterning and xerobranching in the *Arabidopsis *root tip.** A** Hydropatterning regulates lateral root development during prebranch site formation at the transition domain [48]. The contact (water) side follows canonical auxin-dependent founder cell specification and subsequent lateral root initiation [5] at the protoxylem pole (xylem indicated by X at the top of the root). On the air side, canonical auxin signaling is blocked by translational modification (SUMOylation, indicated by a magenta dot) of the transcription factor ARF7, resulting in a stable repression by IAA3 and arrested lateral root initiation [39]. In parallel, on the dry side, there is a biased synthesis of ethylene, which results in inhibition of lateral root formation [40]. **B** Unlike hydropatterning, in xerobranching, phloem-delivered ABA accumulates at the root tip when roots grow through an air gap, inhibiting lateral root development from the prebranch formation stage onwards [7]. ABA is transported from the vascular tissues outwards, resulting in the closure of plasmodesmata, thus limiting auxin diffusion from the outer tissues to the internal pericycle cells [3]. Growth in an air gap also triggers the accumulation of ROS in the root tip, especially in nuclei, resulting in multimerization of the auxin signaling repressor IAA3 and increasing its efficiency as a transcriptional repressor [47]. Note that root schematics show an extra portion of root corresponding to the early differentiation zone (distinguished by the presence of root hairs) where lateral root primordia initiate. Root cross section adapted from De Smet et al. [35]

Roots are acutely responsive to spatiotemporal variation in water availability and capable of developmental responses that increase access to water. Hydrosignaling is a broad term describing the signal-transduction pathways that mediate moisture responses [3]. Responses to moisture have been distinguished by researchers based on the environmental context and the developmental process that is controlled. Hydrotropism, first documented by Charles and Francis Darwin [4], describes the root tip’s ability to perceive moisture gradients, either in air or in the surrounding growth substrate, and steer root growth toward greater water availability (Fig. 1). Hydropatterning, in contrast, was described more recently [5]. This process requires direct contact between root tissues and water-conductive surfaces, leading to the localized induction of root branches in contacting tissues, while branch formation in air-exposed tissues is suppressed. This response is also perceived at the root tip [5, 6] but leads to developmental changes outside of the growth zone. Described most recently, xerobranching involves the complete suppression of root branching when a root grows into air spaces such that the tip completely lacks contact with a water-conducting surface [7]. Xerobranching is distinguished from hydropatterning due to its dependence on the water-deficit stress-associated hormone, abscisic acid (ABA), which is synthesized in the phloem and transported to outer tissues to inhibit lateral root initiation [3] (Fig. 2).

The exploration of developmental responses to hydrosignaling has revealed the role of different signaling pathways that act downstream of moisture perception. Our understanding of how root cells initiate hydrosignaling—by perceiving the physicochemical changes associated with water availability—remains limited. Recent work suggests that indirect proxies for water status, such as air-filled soil spaces that trap the hormone ethylene, may underlie moisture-dependent responses [8]. In this review, we first describe new insights into hydrosignaling that have clarified how various downstream developmental processes are controlled. Following this, we explore the direct and indirect mechanisms involved in the perception of these moisture cues. Together, this work highlights the sophisticated mechanisms plants employ to perceive spatiotemporal variation in water availability while identifying molecular components that serve as promising targets for enhancing agricultural sustainability.

### Hydrotropism: moisture-regulated root tip growth

Directing root growth toward moisture during hydrotropic stimulation requires differential growth and appears to involve tissues that are distinct from gravitropism, growth in response to the gravity vector. Kinematic analysis of hydrotropically stimulated maize root tips revealed differential growth between the so-called wet and dry sides of the root, with wet-side tissues showing the largest drop in growth rate compared to similar tissues in roots grown under uniform-moisture conditions [9]. In *Arabidopsis* (*Arabidopsis thaliana*) and rice (*Oryza sativa*), laser removal of the root apical meristem and root cap, which are tissues critical for gravity response, does not abolish hydrotropic bending, suggesting that perception and response of the hydrotropic cue lie elsewhere [10, 11]. These data and others highlight the importance of the transition zone, which is the region of the root tip at the boundary of the meristematic and elongation zones (hereafter “the transition/elongation zone”), as the most important region for sensing and responding to environmental moisture gradients.

At the molecular and cellular levels, the role of the drought-responsive hormone, abscisic acid (ABA), has been extensively documented in hydrotropism, though the exact function appears to vary by species. In *Arabidopsis*, while evidence for differential accumulation is currently lacking, ABA-mediated signaling is required in the cortex to control differential cell elongation through the kinase SnRK2.2 (Fig. 1). Targeted expression of *SnRK2.2* in cortex cells, but not in other tissues, rescues the hydrotropic defect in the *snrk2.2 snrk2.3* double mutant [10]. Interestingly, expression of the hydrotropism regulator MIZU-KUSSEI1 (MIZ1) is enriched in cortex cells, where it is also required to promote the hydrotropic response (Fig. 1) [10, 12]. In tomato (*Solanum lycopersicum*), ABA was measured to asymmetrically accumulate in the root tip, likely due to elevated biosynthesis in tissues facing the lower water-potential media, and defects in ABA biosynthesis strongly disrupted hydrotropic bending [13]. In maize (*Zea mays*) roots, ABA levels rise after hydrotropic stimulation; however, there is no evidence of differential accumulation between the “wet” and “dry” sides of the root [9]. ABA can have both growth-inhibitory and stimulating effects that vary between species, which likely underlies the varying contribution of this hormone to hydrotropism [14, 15].

Calcium signaling is another key component mediating the hydrotropic response. In gradients of water potential (Ψ), a measure of water’s free energy relative to a sample of pure water at rest, cytosolic Ca^2^⁺ levels rise in the root tip and peak in the elongation zone ~ 40 min after stimulation (Fig. 1). The Ca^2^⁺ signal travels slowly through the phloem, compared to other reported calcium dynamics in plants that range from seconds to a few minutes [16–20]. At the transition/elongation zone of the root tip, the transient Ca^2^⁺ signal peak disperses asymmetrically and laterally with a higher signal on the convex side of the bending root tip. The cytoplasmic Ca^2^⁺ release depends on inhibitory interactions between MIZ1, which encodes an ER-associated membrane protein, and the endoplasmic reticulum (ER) Ca^2^⁺-ATPase ECA1 [21]. ABA signaling often leads to transient increases in cytosolic calcium levels, and recent work revealed that ECA1 plays an important role in reducing the amplitude of ABA-mediated osmotic stress responses by sequestering calcium back into the ER [22]. It will be interesting to determine whether hydrotropism-mediated changes in calcium levels are ABA dependent and whether phloem-dependent delivery of ABA, which occurs during xerobranching [3], also plays a role in hydrotropism.

The direction of greatest moisture may diverge from the gravity vector; thus, roots must decide which environmental gradient to follow. ABA signaling during the hydrotropic response activates phospholipase Dζ2 (PLDζ2) and expands its expression from the transition zone to the apical meristem, including the root cap, where it suppresses gravitropic responses [23]. This mechanism is accompanied by autophagic degradation of statoliths in the root cap [24, 25] and at the transition/elongation zone where curvature occurs [26].

Other signals are also involved in the antagonistic relationship between hydrotropism and gravitropism. Hydrostimulation attenuates asymmetries in the distribution of auxin, a key hormone for plant development, and the reactive oxygen species (ROS) that are normally induced by gravistimulation [27, 28]. Specific transport inhibitor response 1 (TIR1)-dependent auxin signaling antagonists (PEO-IAA and auxinole) accentuate hydrotropic bending without inducing asymmetric auxin distribution. Treatment of roots with the polar auxin transport inhibitor NPA resulted in a similar outcome to the auxin signaling antagonists [27]. These data indicate that auxin negatively regulates hydrotropism, and that the hydrotropic response is independent of PIN-Formed (PIN) efflux carrier-mediated auxin transport and TIR1-dependent signaling, at least in *Arabidopsis*. Interestingly, auxin signaling, transport, and synthesis do seem to play an important role in the hydrotropic response in other species [11]. While auxin transport and response inhibitors reduced hydrotropic and gravitropic responses in rice, they only reduced hydrotropism in *Pisum sativum* roots and reduced gravitropism but enhanced hydrotropism in *Lotus japonicus* [11]. Auxin redistribution is also observed in hydrostimulated maize root tips prior to bending [9], suggesting that it may have a more instructive role in this species. Other hormones, such as salicylic acid and cytokinin, showed differential accumulation between the wet and dry sides of maize root tips after hydrostimulation, with higher levels of both hormones on the wet side [9]. In *A. thaliana*, the cytokinin hormone pathway is also involved; however, hydrostimulated roots exhibit higher signaling activity on the dry side of the root, leading to increased cell proliferation that is important for promoting root tip bending downstream of *MIZ1* [29].

More recently, it has been demonstrated that MIZ1’s role in hydrotropism may primarily center on its regulation of the gravitropic response [30]. To better untangle the relationship between these two tropisms, the authors utilized a clinostat, a mechanical device that rotates plants growing on petri dishes at a specific speed to counteract the force of gravity. Surprisingly, *miz1* still exhibited a hydrotropic response under these conditions. These data suggest that MIZ1’s primary function is to suppress the response to gravity, while the tropic response to moisture is independent of it. Consistent with this hypothesis, disruption of the auxin-transport pathway responsible for gravitropism rescued the hydrotropic defect of the *miz1* mutant. The authors also show that MIZ1 has a more general role in suppressing gravitropism when seedlings are exposed to low water potential conditions that are non-directional. Sorbitol suppresses gravitropism by inhibiting gravity-induced PIN3-GFP repolarization in wild type; however, this response is blocked in *miz1*. These data show that in *Arabidopsis*, osmotic stress induces a MIZ1-dependent negative regulation of gravitropism that is mediated through the control of PIN protein polar relocalization (Fig. 1). These findings also suggest that additional hydrosignaling components are likely necessary to induce hydrotropic bending since this response can still occur in a *miz1* background.

At the organ system scale, drought induces ABA-dependent auxin synthesis, which changes the gravitropic set-point angle, resulting in steeper root systems in rice and maize [31]. The ABA biosynthesis mutant *Osmhz5* develops markedly shallower root systems under drought, despite producing a normal number of lateral branches. This phenotype arises from impaired gravitropism, which in *Osmhz5* is associated with a failure to elevate auxin levels under drought; notably, treatment with exogenous auxin rescues the defect. *OsMHZ5* encodes a carotenoid isomerase required for ABA biosynthesis and also functions downstream of ethylene signaling, with *mhz5* mutants showing reduced ethylene responsiveness in roots [32, 33]. An open question is whether this ABA–ethylene–auxin relationship similarly regulates the gravitropic set-point angle during drought. In *Arabidopsis* rhizotron studies, water deficit increases the gravitropic response of recently emerged lateral roots near the soil surface, deepening the root system [34]. Interestingly, *tir1* mutants are impaired in this response and remain shallow under drought, whereas *miz1* behaves similarly to wild type and develops deeper roots, implying distinct regulation of hydrotropism and drought-induced changes in gravitropic set-point control.

### Hydropatterning: spatial regulation of branching and root anatomy when water is heterogeneously distributed

Hydropatterning refers to the spatial regulation of lateral root formation and tissue differentiation—including root hairs, aerenchyma, and exodermis—in response to local differences in water availability along the circumferential axis of the root (Fig. 2A) [5, 36, 37]. Based on the analysis of early lateral root development markers, hydropatterning has been shown to act at or before the founder cell specification stage [38], setting the circumferential position of new primordia. Contact of roots with a wet surface locally induces markers of auxin biosynthesis (*TRYPTOPHAN AMINOTRANSFERASE OF ARABIDOPSIS 1*, *TAA1*), auxin transporters (*PIN-FORMED 3*, *PIN3*), and induces auxin signaling, suggesting that this hormone pathway plays a predominant role in promoting the downstream developmental responses to higher moisture availability [5]. Biased activation of the auxin signaling pathway has been observed in *Arabidopsis*, rice, and maize [5], suggesting that it may act as a conserved mechanism for moisture-induced lateral root formation across flowering plants. ABA is a signal typically associated with water deficit; however, a high-order ABA receptor mutant (*pyr/pyl 112,458*) exhibits normal hydropatterning, suggesting that the suppression of lateral roots in air-exposed tissues is likely independent of this signaling pathway [5].

Posttranslational control of auxin signaling further refines hydropatterning [39]. SUMOylation of AUXIN RESPONSE FACTOR 7 (ARF7), an auxin-response transcription factor necessary for lateral root development, blocks lateral root initiation on the air-exposed side of the primary root. Brief air exposure rapidly increases SUMOylated ARF7. Additionally, the Aux/IAA repressor IAA3/SHY2 harbors a SUMO interaction domain, and a mutation in that domain prevents its interaction with SUMOylated ARF7. While LBD16 shows enriched expression on the contact side of the primary root, ARF7 does not; instead, ARF7 SUMOylation on the air side recruits the repressor IAA3/SHY2, preventing LBD16 expression and thus lateral root initiation there. Non-SUMOylated ARF7 on the contact side activates *LBD16* and triggers initiation. How differential exposure to moisture locally determines the activity of the SUMOylation pathway remains to be determined.

Of the various developmental responses of roots to moisture, few studies have examined the degree to which these responses vary across natural accessions or crop varieties. To facilitate large-scale screening for variation in hydropatterning, Scharwies et al. [40] established a growth system to create a moisture gradient around the primary root tip of maize. Characterization of a panel of 250 diverse inbred lines revealed that most lines exhibit strong hydropatterning; however, inbreds with a weaker response tend to occur in specific subpopulations of maize. Tropical–subtropical lines, for example, show stronger hydropatterning than temperate lines, and quantitative assessment of selection pressure suggests these differences arose due to divergent selection.

GWAS/TWAS in maize, and additional functional validation in *Arabidopsis*, identified several loci contributing to hydropatterning [40]. Consistent with the importance of auxin signaling in mediating moisture-induced lateral root development on the contact side, TWAS analysis linked higher *ZmAXR1* expression to increased air-side branching, likely via enhanced auxin sensitivity. Conversely, mutations in *AtFLA4*, a negative regulator of ethylene biosynthesis, enhanced the hydropatterning response, leading to a reduction in air-side lateral roots. Subsequent analysis using pharmacological and genetic perturbations in ethylene biosynthesis and signaling showed that ethylene, rather than the precursor ACC, acts on the air side to suppress lateral root development. A working model proposes that the root senses heterogeneity in the availability of water through two mechanisms: in air spaces, the biosynthesis of ethylene suppresses air-side branching, while contact of root tissues with a moist surface locally promotes auxin signaling and transport pathways to induce contact-side branching.

### Xerobranching: a response to the complete lack of available water

Xerobranching, distinct from hydropatterning, describes the complete suppression of lateral root formation when roots lose all contact with moist soil and traverse large air spaces, causing transient local water deficit (Fig. 2B) [7]. X-ray microCT imaging of maize and barley (*Hordeum vulgare*) showed that lateral root development is arrested when roots cross ~ 2-cm air gaps (macropores). Transcriptomics of affected barley segments identified regulatory modules involving CK, ABA, auxin, salicylic acid, and jasmonic acid. Unsurprisingly, given its central role in drought responses, quantification of ABA revealed ~ fourfold higher levels in roots growing in air spaces. Exogenous ABA treatment in aeroponics produced discrete, non-branching segments in barley and maize, while growth of plants on solid media supplemented with ABA in *Arabidopsis* and barley yielded similar repression. Time-lapse imaging of the *DR5::luciferase* auxin reporter in *Arabidopsis* showed that ABA disrupts the normal temporal oscillations occurring at the tip, preventing pre-branch site establishment; in maize, DR5 reporter activity likewise diminished upon ABA treatment, and IAA levels decreased in maize tips. Thus, xerobranching represses branching at very early stages through an ABA-related mechanism [7].

Definitive evidence that ABA is a key regulator of xerobranching was provided by Mehra et al. [3], who identified a mechanism linking hydraulic changes in the root to shifts in the dynamic distribution of ABA and auxin, which control lateral root branching [3]. In growing root tips, water required for growth is supplied by the external environment as well as by the phloem [41, 42], which differentiates close to the root tip [43]. Roots growing into air pockets lose their external source and must rely upon the phloem entirely. Perception of water deficit leads to the release of ABA from the phloem [3], as shown using the ABACUS2 ABA FRET sensor [44]. ABA induces the deposition of callose at the plasmodesmata, which may restrict symplastic diffusion of auxin from the outer to the inner tissues. This restriction in auxin diffusion may prevent the transmission of the auxin cue that is normally released from the lateral root cap to induce founder cells in the inner pericycle tissue [45, 46].

Subsequent work has placed ROS upstream of ABA in xerobranching. In roots entering macropores, nuclear ROS spikes, dependent on RBOH enzymes, precede ABA accumulation [47]. *RBOH* mutants continue branching under xerobranching stimulation, and *RBOHB/E/F* expression increases upon treatment. An H₂O₂ sensor (*roGFP2-Orp1*) revealed strong signals in elongation zone nuclei of roots crossing air gaps. ROS triggers multimerization of IAA3 via disulfide-bond formation at cysteine residues, thereby repressing lateral root initiation. The *IAA3* knockout mutant shows defective xerobranching, while a mutant version of IAA3 lacking four cysteine residues failed to multimerize, failed to repress lateral root initiation, and exhibited weakened interactions with the co-repressor TOPLESS.

## Putative mechanisms of moisture perception

Signal transduction cascades most commonly involve three components: a signal, a receptor, and a response. So far, only the response piece of the interaction between roots and water is understood with any appreciable clarity. As reviewed in previous sections, a growing body of work suggests that plant hormones [40], reactive oxygen species [47], and posttranslational modification pathways [39, 47] play important roles in regulating root architecture as a response to moisture or absence thereof. The search for signals and receptors is ongoing. It remains unclear how water produces the signaling cue and which component of the plant cell is responsible for perception. This section reviews recent efforts to identify such perception modules in roots, drawing insights from studies in the model plant *Arabidopsis*, in crop species, and from knowledge of moisture-sensing mechanisms in non-plant organisms across the Tree of Life.

### The role of mechanical cues in perception of water availability

**Fig. 3:**
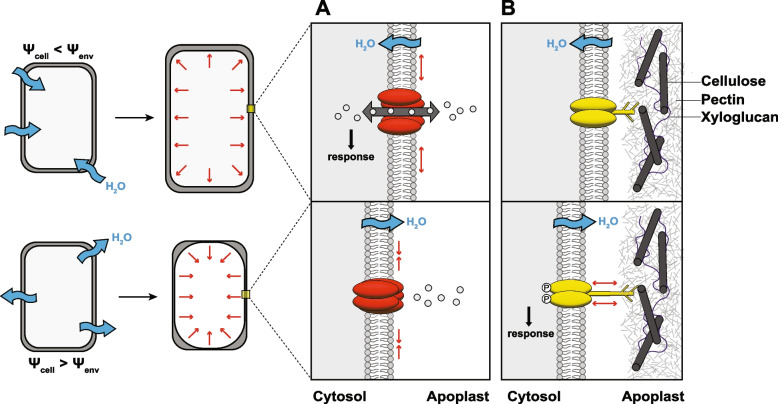
Putative mechanosensors for perception of water availability in plant cells. Water uptake (top row) and water loss (bottom row) lead to changes in cell volume and generate mechanical forces at the plasma membrane–cell wall interface. **A** Mechanosensitive ion channels (colored red) are activated by tension (red arrows) in the plasma membrane. Reduced tension from turgor loss causes closure of the channel. **B** Membrane proteins with extracellular domains (i.e., receptor-like kinases, colored yellow) may sense physical cues, like separation of the wall–membrane interface during plasmolysis and turgor loss, and convert these into chemical signals, such as via phosphorylation

Hydropatterning competency depends on cell growth [6], which requires water uptake from the environment and careful control of the cell’s volume and hydrostatic pressure. How cells perceive their internal volume in a changing environment is poorly understood. Mechanical forces generated at the plasma membrane and the cell wall during water uptake, turgor fluctuations, or plasmolysis represent potential sources of mechanical stimulation that could trigger a signal transduction cascade. Proteins with extracellular or transmembrane domains like receptor-like kinases may be able to convert these hydraulic cues into chemical signals (Fig. 3).

Mechanosensitive (MS) ion channels are required for humidity sensing in some animals [49, 50], and a number of orthologs have been suggested to play similar roles in plants [51–53]. For example, the MscS-Like ion channel MECHANOSENSITIVE CHANNEL OF SMALL CONDUCTANCE-LIKE 10 (MSL10) was shown to regulate cell swelling during hypoosmotic stress [54]. The authors established a cell swelling assay for *A. thaliana* seedlings and showed that MSL10 over-expression lines exhibited increased levels of cytosolic Ca^2+^ and reactive oxygen species (ROS), two documented markers of the hypoosmotic stress response, as well as higher expression of known mechano-inducible genes. Investigations into MSL10 and its nine homologs are ongoing in *Arabidopsis*. Recent studies suggest MSL10 is particularly responsive to pulsed (0.3–3 Hz) mechanical forces like those generated by wind [55] and associates with proteins at endoplasmic reticulum-plasma membrane contact sites (EPCSs) [56], a localization uniquely positioned to transduce signals across the cell boundary.

Calcium is maintained in millimolar concentrations in the apoplast and rapidly transported into the cell in response to a variety of stimuli, such as pathogen infection, touch, and osmotic shock [57]. The role of [Ca^2+^] as a secondary messenger has long prompted a search for plant Ca^2+^ channels responsive to specific cues, with the assumption that such sensing constitutes the initial signaling event. A number of mechanically gated calcium channels have been identified; for example, the stretch-activated MID-COMPLEMENTING ACTIVITY 1 (MCA1) and PIEZO1 (PZO1) Ca^2+^ channels, which are required for *Arabidopsis* roots to penetrate hard agar [58, 59]. Another candidate channel, REDUCED HYPEROSMOLALITY-INDUCED Ca^2+^ INCREASE (OSCA1), was identified as an osmosensitive (OS) Ca^2+^ influx channel [60] and shown to negatively regulate hydrotropism in *Arabidopsis* seedlings [61]. Interestingly, these findings appear to conflict with a previous model that transient increases in [Ca^2+^]_cyto_ are required for hydrotropism [21] and may suggest a role for calcium in attenuating moisture responses rather than driving them directly.

The role that these candidate osmosensitive genes play in hydrosignaling remains to be determined. However, it should be noted that mechanosensitive ion channels are unlikely to be the sole mechanism of moisture perception in the plant root. Mutant alleles of these genes, including a *msl4-1; msl5-2; msl6-1; msl9-1; msl10-1* quintuple mutant, typically result in subtle and nonlethal phenotypes [62]. Perhaps plant moisture sensing involves several mechanisms of perception in parallel, as it does in animals [49, 63]. Future studies should continue to explore a diversity of potential signaling pathways and be wary of overinterpreting the results of mechanosensing experiments, which are often conducted in idealized physical systems focused on narrowly defined responses [64].

### Biomolecular condensates: an emerging paradigm in environmental perception

Biomolecular condensates are membraneless cellular compartments containing specific macromolecules (i.e., proteins and nucleic acids) that can form transiently in response to stimuli. A growing number of studies suggest that biomolecular condensates play important roles in water perception and stress response. For example, the plant transcriptional regulator SEUSS (SEU) confers osmotic stress tolerance by forming nuclear condensates in response to hyperosmotic shock [64]. This effect appears to be driven by liquid–liquid phase separation, requiring the SEU disordered N-terminal domain to drive condensation and rescue the survivability of *seu* mutants challenged with mannitol [65]. Several other hydration-dependent condensation events have been documented in *Arabidopsis*, namely, in the prion-like FLOE1 and the RAF12 kinase [66–68]. FLOE1 regulates seed germination, a process tightly linked with perceiving seed hydration status, while RAF12 and its signaling partners orchestrate root response to osmotic stress. Both FLOE1 and RAF12 functions were found to be contingent on the ability of the proteins to form reversible condensate structures via their disordered domains. Proteins lacking a fixed three-dimensional structure are often highly sensitive to the physicochemical environment of the cell by virtue of their exposed residues and are emerging as *bona fide* sensors of these stimuli. Recently, the tendency of LATE EMBRYOGENESIS ABUNDANT 4–5 (LEA4-5) to adopt a folded structure under water deficit conditions was leveraged to develop a molecular-crowding sensor responsive to changes in hydration state [66, 69].

### The cell wall as a sensing interface for water availability

The plant cell wall is a hydrated matrix of polysaccharides that forms an interface between cells and their environment. Changes in cell wall integrity (CWI) are closely monitored, for example, during growth, when cells must elongate while avoiding mechanical failure, and represent a source of signals that regulate development, immunity, and stress responses [70]. However, how the properties of the wall, up to 80% water by weight, shift with water availability remains understudied, as are the potential mechanisms to sense such changes. Mechanical studies suggest that hydration states correlate with bulk moduli of polysaccharide films [71] and the extensibility of cell-wall microsections [72]. In the material sciences, it is well established that the properties of synthetic polymer matrices can be modulated by the embedding of plasticizers—low-molecular-weight spacer molecules [73]. Might water play similar roles in the plant cell wall? In sunflower hypocotyls, the cell wall’s extension rate decreased when water potential was reduced to − 0.62 MPa by treatment with polyethylene glycol 6000 (PEG), a high-molecular-weight osmolyte that cannot penetrate the cell-wall matrix [74]. The same effect was observed, albeit more subtly, in cell-wall preparations reconstituted in vitro from extracts of bacterial cellulose, apple pectin, and tamarind xyloglucan. In situ measurements of cell-wall hydration and growth dynamics, like those enabled by the biosensor AquaDust [75], are needed to corroborate these in vitro studies. Until then, the cell wall remains a mysterious biochemical environment and its role in guiding developmental responses to moisture has yet to be thoroughly examined.

### Do roots perceive water through organ-scale sensing mechanisms?

The perception mechanisms reviewed thus far have assumed the involvement of direct signals—mechanical or osmotic forces, molecular crowding, and cell-wall extensibility—and the existence of receptors attuned to those signals (Fig. 3). Another possibility that is not mutually exclusive is that moisture sensing is achieved indirectly at a *system* level, much in the same way that roots perceive soil compaction. In brief, plants perceive soil compaction not solely through mechanical forces but also by sensing elevated levels of ethylene—a growth-inhibiting hormone produced in roots that accumulates as its diffusion through compacted soil is restricted [8]. Experiments performed in rice and *Arabidopsis* showed that compacted soils were more likely to accumulate ethylene, leading to increased ethylene signaling in the root and thus less growth. In this model, the signal (ethylene) is produced endogenously and emitted into the environment. The structure of the pore spaces in the soil modulates this cue, capturing it near the root when soils are compacted while allowing it to diffuse away when soils are porous. The root responds to elevated ethylene by inhibiting growth, a response well attuned to avoiding root investment in compacted soil. Might moisture-sensing occur via a similar mechanism? We consider two examples below.

### The “sense-by-growth” hypothesis

**Fig. 4:**
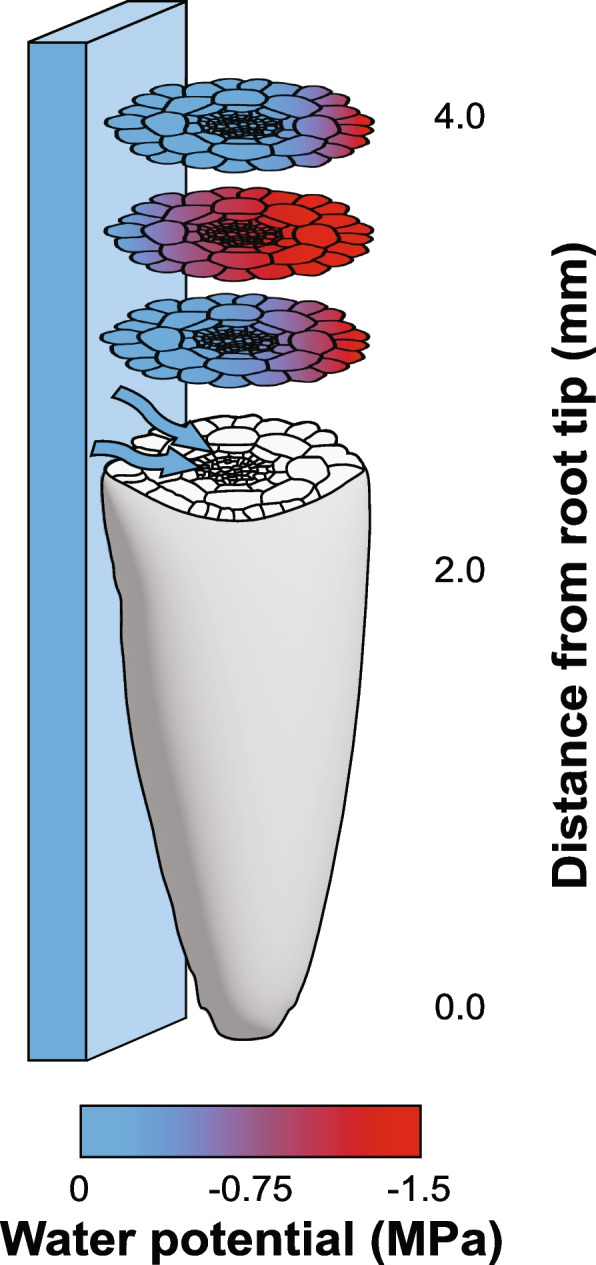
Modeling-based prediction of hydraulics in the root tip. Estimated tissue water potentials in a growing *Arabidopsis* root are based on a computational model in Robbins and Dinneny [6]. Blue arrows indicate the direction of water diffusion. The gray portion represents the surface of the root tip. The water potential in internal root tissues is indicated as a blue–red gradient pattern. The blue box next to the root represents a moist surface in contact with the root

For hydropatterning, evidence suggests that direct sensing of a water potential differential between the root and environment is unlikely since stark differences in root branching can be observed even under conditions where the air and media are at water-potential equilibrium [5]. Perhaps the rate of water uptake from the environment into plant tissues represents a systemic signal that triggers hydropatterning instead, as roots contacting water-conductive materials (soil, agar and paper) induce branching while nonconductive materials (plastic, glass, rubber) do not. In maize, perception of the moisture gradient driving hydropatterning may rely on a “sense-by-growth” mechanism localized to the first ~ 5–6 mm of the root tip. Computational modeling of tissue hydraulics and experimental manipulation of growth showed that water uptake from expanding cells near the root tip can generate a water potential gradient across the root diameter if water is heterogeneously distributed in the environment (Fig. 4) [6]. Results from the model also suggest that gradients of tissue water potential are strongly predictive of lateral root patterning. Though the model falls short of explaining how such internal water potential gradients are perceived, it suggests the physiological status of a cell is likely important for determining what properties of water availability can be perceived.

Technical challenges in measuring cell-scale water potentials of live tissues have prevented evaluation of some of the model’s predictions. Promising noninvasive methods such as in situ cavitation bubble manometry and FRET-based sensors may permit measurements of water potential in live tissues but have yet to be thoroughly investigated in plant systems [69, 76].

### Does ethylene act as a positional cue for air spaces in soil?

**Fig. 5:**
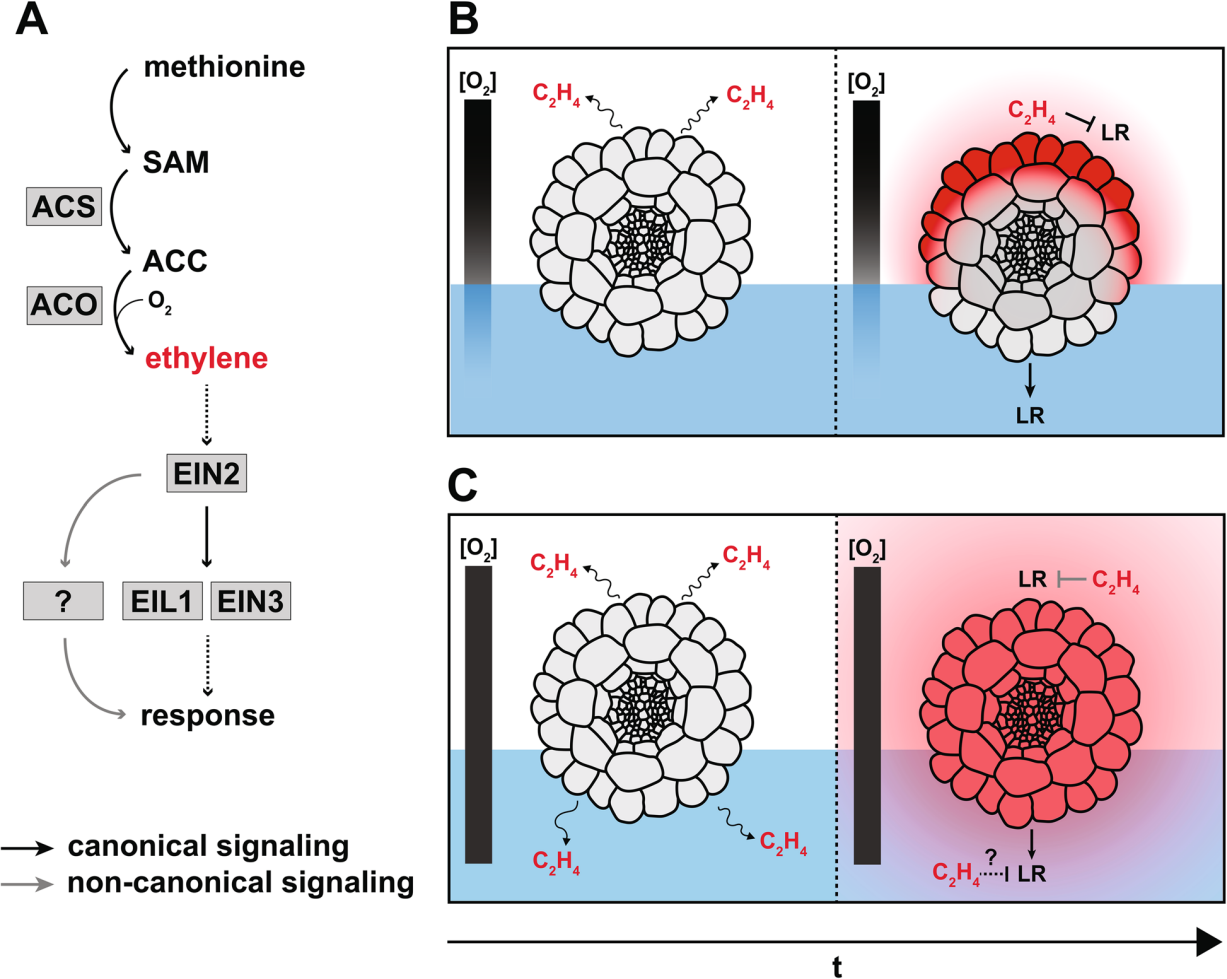
Ethylene-mediated hydropatterning models.** A** Ethylene biosynthetic pathway and simplified downstream signaling response. Enzymes and genes are represented in gray boxes. **B** Ethylene (in red) mediates air-side repression of LR formation under limited gas diffusion through tissue and the aqueous phase (in blue). **C** Ethylene (in red) mediates air-side repression under homogeneous gas concentrations at the response level via an unknown mechanism. Root cross-section adapted from Dolan et al. *Development* [85]

A role for ethylene in moisture sensing was recently suggested by a genome-wide association study (GWAS) of maize inbred lines [40]. The GWAS identified *FASCICLIN-LIKE ARABINOGALACTAN 4* (*FLA4*)*/SALT OVERLY SENSITIVE 5* (*SOS5*) as a strong negative regulator of hydropatterning. *FLA4/SOS5* encodes an extracellular protein involved in CWI maintenance during osmotic stress events [77, 78]. Though its physical interactors remain elusive, genetic evidence in *Arabidopsis* suggests that *FLA4* inhibits biosynthesis of the ethylene precursor 1-aminocyclopropane-1-carboxylic acid (ACC) through a linear pathway involving the receptor-like kinases FEI1 and FEI2 [79, 80]. Scharwies et al. [40] demonstrated that *fla4* and ethylene-overproducing mutants exhibit enhanced hydropatterning in *Arabidopsis*. Conversely, disrupting ethylene synthesis or perception resulted in increased air-side lateral root density. This raises an important question: how can ethylene selectively inhibit lateral root formation on the air side, and not the contact-side? We propose two potential models: (1) ethylene biosynthesis from cells directly contacting air generates an internal concentration gradient, with higher air-side ethylene levels near the site of synthesis inhibiting lateral root development in air-exposed tissue; (2) alternatively, ethylene may diffuse freely across and around the root axis, leading to a concentration equilibrium over time. In the second model, a side-specific response is triggered at the level of ethylene signal transduction (Fig. 5).

In support of the first model, measurements of oxygen, which is required for ACC conversion into ethylene, revealed sharp radial concentration gradients in the maize root [81]. If diffusion rates can limit gas distribution across tissues < 1 mm in length, perhaps the highest concentration of ethylene is found in air-exposed epidermal cells, where oxygen-dependent synthesis can occur.

In the second scenario where air-side specificity of ethylene’s lateral root suppression is determined downstream of ethylene perception, it should be noted that a noncanonical signaling pathway is likely involved, since a double mutant of ETHYLENE-INSENSITIVE3 (EIN3) and ETHYLENE-INSENSITIVE-LIKE 1 (EIL1), *ein3/1eil1*, two transcriptional regulators known to mediate response to ethylene, showed no change in air-side lateral root density compared to wild-type *Arabidopsis* [40]. Among possible candidates for pathways that could gate ethylene response are calcium signaling [21] and other hormone signaling pathways. Xerobranching, for instance, appears phenotypically similar (though more extreme) to the hydropatterning response but is regulated predominantly by the hormone ABA [7], itself a suggested regulator of ethylene synthesis [82, 83]. Hence, hydropatterning may be the product of a signaling network involving multiple hormones that cross-talk to mediate root branching [84].

## Conclusions

The soil environment, which roots must explore to uptake water and nutrients, is a complex setting where resource distribution varies widely in both space and time. Roots have evolved developmental mechanisms to navigate this heterogeneity. We have seen that three such strategies—hydrotropism, hydropatterning, and xerobranching—allow root systems to pattern their architecture in response to moisture cues. Far from redundant, these strategies seem to operate via distinct signaling pathways, across different tissue layers, and in response to various water availability scenarios. So far, these processes have been studied largely in isolation. Still, over the last decade, considerable progress has been made in characterizing their underlying genetics and modeling testable hypotheses for perception at the organ scale. However, whether the three moisture cue-dependent responses connect through an underlying signaling network must be further explored. The intriguing rise in phloem calcium levels observed during hydrotropism and the importance of phloem-delivered ABA for xerobranching suggest the potential for cross talk across these pathways. Also missing from our understanding are the early molecular triggers for these responses and an integrated view of the effects of hydrotropism, hydropatterning, and xerobranching on organism fitness and crop performance in the field. Given the dwindling supply of freshwater available to agriculture and land-based life, a richer picture of plant-water relations is urgently needed. Roots are ancient solutions to life’s search for water; their innate moisture sensitivity may help us imagine the future of living systems on a changing planet.

## Data Availability

Not applicable.

## Glossary

Differentiation/maturation zone: The region of the root located above (shootward) the elongation zone, where cells complete their differentiation. Differentiated tissues (i.e., Casparian strip and root hair cells) establish their specialized anatomical features, and lateral roots initiate their growth in this zone.
Elongation zone: The region of the root located shootward of the division zone, where dramatic cell expansion occurs. Cellular elongation is a component of root growth.
Founder cells: Reprogrammed cells committed to the initiation of a new organ primordium. In *Arabidopsis*, lateral root founder cells are specified in xylem-pole pericycle cells and divide to initiate lateral root primordium development.
Gravitropism: It is a directional growth in response to gravity. Roots exhibit positive gravitropism, growing in the direction of the gravity vector, while shoots typically exhibit negative gravitropism.
Hydropatterning: Biased lateral root formation and tissue differentiation in relation to the contact of the parent root with moist surfaces and areas of high water availability.
Hydrosignaling: A broad term used to describe the signaling pathways mediating organ-scale responses to variation in water availability (i.e., hydropatterning, hydrotropism, xerobranching).
Hydrostimulation: A broad term used to describe a moisture cue (i.e., a water potential gradient) that can trigger a response (i.e., hydrotropism).
Hydrotropism: A growth response to a moisture gradient. Roots typically exhibit positive hydrotropism: growth *toward* a water source.
Primordium/primordia: The earliest stage of an organ in development before becoming a proper organ. It is often abbreviated as LRP (lateral root primordium) in lateral root formation where it initiates from pericycle cells and undergoes rounds of cell division to produce a dome-shaped structure that eventually emerges from the parent root.
Meristematic zone/root apical meristem: A distal region of the root tip where cells are actively undergoing mitosis, producing new cells for the rest of the root system. A cluster of undifferentiated stem cells is maintained within the meristem, which continuously produces daughter cells giving rise to different cell types (see Fig. 1A). Alongside the elongation zone, the root apical meristem also contributes to root growth.
Root cap: It is the most distal portion of the root tip that serves as a protective tissue, shielding the meristematic zone, including the stem cell niche, from mechanical damage as the root pushes through the soil. Root cap cells are continuously shed and produced to maintain a stable cap size. The root cap also plays a role in sensing and signaling.
Root tip: Although the meaning may be ambiguous across the literature. By root tip, here we mean the distal portion of a root that includes the root cap, the meristematic zone, and the early elongation zone.
Xerobranching: A temporary suppression of lateral root formation in response to the loss of contact of the root tip and moisture, for example, when the root encounters a large air gap.
